# The Landscape of Autophagy-Related (ATG) Genes and Functional Characterization of *TaVAMP727* to Autophagy in Wheat

**DOI:** 10.3390/ijms23020891

**Published:** 2022-01-14

**Authors:** Wenjie Yue, Haobin Zhang, Xuming Sun, Ning Su, Qi Zhao, Zhaogui Yan, Song Weining, Hong Yue

**Affiliations:** 1 State Key Laboratory of Crop Stress Biology in Arid Areas, College of Agronomy, Northwest A&F University, Xianyang 712100, China; ywenjie993@nwafu.edu.cn (W.Y.); zhb626@nwafu.edu.cn (H.Z.); SunXM@nwafu.edu.cn (X.S.); sunnying@nwafu.edu.cn (N.S.); zhaoqi1204@nwafu.edu.cn (Q.Z.); 2 College of Horticulture and Forestry Sciences, Huazhong Agricultural University, Wuhan 430070, China; gyan@mail.hzau.edu.cn

**Keywords:** autophagic homeostasis, Triticeae species, abiotic stress, seed size, *TaVAMP727*, evolution

## Abstract

Autophagy is an indispensable biological process and plays crucial roles in plant growth and plant responses to both biotic and abiotic stresses. This study systematically identified autophagy-related proteins (ATGs) in wheat and its diploid and tetraploid progenitors and investigated their genomic organization, structure characteristics, expression patterns, genetic variation, and regulation network. We identified a total of 77, 51, 29, and 30 ATGs in wheat, wild emmer, *T. urartu* and *A. tauschii*, respectively, and grouped them into 19 subfamilies. We found that these autophagy-related genes (*ATGs*) suffered various degrees of selection during the wheat’s domestication and breeding processes. The genetic variations in the promoter region of *Ta2A_ATG8a* were associated with differences in seed size, which might be artificially selected for during the domestication process of tetraploid wheat. Overexpression of *TaVAMP727* improved the cold, drought, and salt stresses resistance of the transgenic *Arabidopsis* and wheat. It also promoted wheat heading by regulating the expression of most *ATGs*. Our findings demonstrate how *ATGs* regulate wheat plant development and improve abiotic stress resistance. The results presented here provide the basis for wheat breeding programs for selecting varieties of higher yield which are capable of growing in colder, drier, and saltier areas.

## 1. Introduction

Autophagy is an evolutionarily conserved intracellular vacuolar process that controls recycling cellular contents and organelles to promote cell survival and redistribute nutrients. As a highly conserved intracellular degradation system, autophagy is believed to be responsible for the self-defense and protection of plants from biotic and abiotic stress. Three types of autophagy, microautophagy, macroautophagy, and mega-autophagy, have been identified in plants [1]. The role of autophagy in plants is paradoxical. On the one hand, it may respond to either stress conditions or nutrient starvation to enable cell survival. On the other hand, autophagy may be associated with programmed cell death (PCD) in extensive cell component degradation [2].

Autophagy is a complex process that involves many autophagy-related proteins (ATGs). It is initiated by the phosphorylation and dephosphorylation of the ATG1/ATG13 complex [3]. ATG9 membrane delivery complex and class III phosphatidylinositol-3-kinase (PI3K) complex are necessary for autophagosome formation [4,5]. Furthermore, ATG8-PE (phosphatidylethanolamine) and ATG5-ATG12 conjugation systems contribute to phagophore expansion and closure [6]. Functions of autophagy in plants might result from the synergy of all autophagy protein complexes, and autophagy in plants might be studied as a whole. Many factors regulate the autophagy of plants. The target of the rapamycin (TOR) complex and sucrose non-fermenting 1-related kinase 1 (SnRK1) are crucial regulators of abiotic stress-induced autophagy in plants [7]. Furthermore, there are considerable overlaps and signaling crosstalks between different cell death pathways and how they are regulated [8]. The soluble N-ethyl-maleimide sensitive factor attachment protein receptors (SNARE) complex is a crucial regulator of vesicular traffic that mediates specific membrane fusion between transport vesicles and target membranes and is essential to autophagy [9]. SNARE proteins could participate in the autophagy process by interacting with many ATGs. In yeast, SNARE proteins mediate the homotypic fusion of Atg9 vesicles. It also bundles to regulate autophagosome-vacuole fusion which is controlled by Atg1 kinase [10,11]. In mammals, both Atg8 and Atg14 proteins could interact with SNARE to regulate lysosome and autolysosome biogenesis [12,13]. SNARE could likely mediate homotypic membrane fusion between vesicles and vacuoles in *Arabidopsis* [14]. Vesicle-associated membrane protein 727 (VAMP727) is a seed plant-specific R-SNARE that mediates vacuolar transport, plant growth, and seed maturation [14,15]. This protein has been reported to play a crucial role in autophagy [16]. However, further studies are needed on the specific functions of VAMP727 in autophagy.

An increasing number of studies have been conducted to reveal the functions of autophagy in both fungi and animals. Similar to yeast, one of the significant physiological roles of autophagy in plants is a cellular adaptation to both nitrogen and carbon starvation [17]. However, it is challenging to use definitions developed in animal and fungi systems indiscriminately since plants may have specific autophagy mechanisms. During the lifespan of plants, autophagy is crucial in plants’ resistance to various biotic and abiotic stresses [18]. Autophagy activation can represent one of the significant responses of plants to deal with these stress conditions. Although several functions of specific autophagy-related genes (*ATGs*) have been studied, the mechanisms on how autophagy regulates plant development or plant responses to biotic and abiotic stresses are largely unknown.

Wheat (*Triticum aestivum* L.) is the most widely cultivated crop worldwide, contributing to about a fifth of humans’ total calorie requirements [19]. Genetically, it is a newly formed allohexaploid species (2n = 6x = 42, AABBDD) that originates from a combination of genomes of three diploid donor species through two naturally interspecific hybridization events [20]. Thus, wheat is an ideal model species for studying chromosome interaction and polyploidization in plants. In recent years, with the completion of genome sequencing and assembly of three wheat-related species, *T. turgidum* ssp. *dicoccoides* (wild emmer), *T. urartu* and *A. tauschii* [19,21,22,23], large-scale wheat resequencing efforts have been carried out to explain the origins and domestication of wheat [24,25]. The *ATGs* in wheat such as *ATG4*, *ATG8* and *ATG6* have been identified as associated with biotic and abiotic stress responses [26,27].

Until now, genome-wide identification and characterization of *ATGs* have been investigated in various plant species. However, the role of *ATGs* in wheat’s abiotic stress resistance and its evolution has not been systematically determined. This study aimed to investigate the function and evolution of the ATG gene family in wheat and its diploid and tetraploid progenitors and to explore the role of *TaVAMP727* in autophagy-related plant growth, development, and abiotic stress responses.

## 2. Results

### 2.1. Identification of ATGs from Wheat and Its Diploid and Tetraploid Progenitors

**Figure 1:**
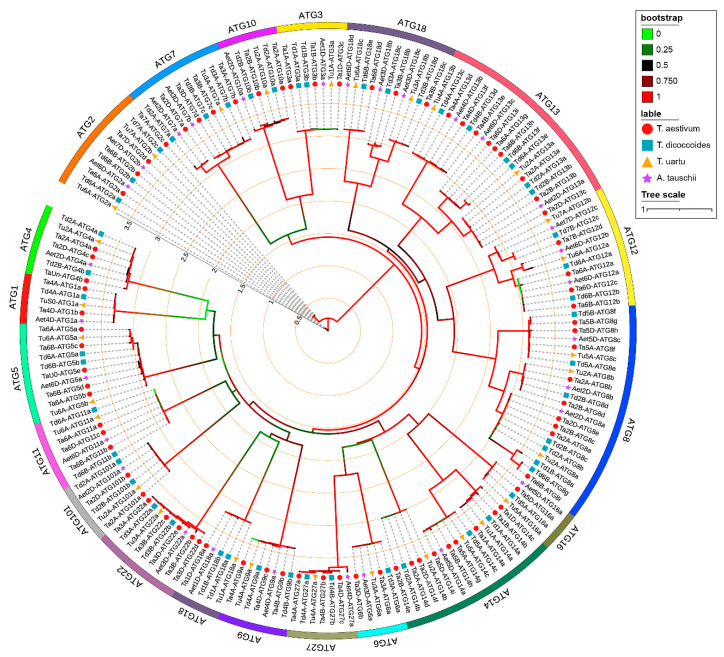
Maximum likelihood phylogeny of autophagy-related proteins (ATGs) from wheat (*T. aestivum*), wild emmer (*T. turgidum* ssp. *dicoccoides*), *T. urartu* and *A. tauschii*. IQ-TREE software was used to construct a phylogenetic tree of ATGs with the bootstrap value estimation based on 1000 iterations and visualized by iTOL. The names with a solid red circle are wheat ATGs, dark-turquoise square are wild emmer ATGs, yellow triangle are *T. urartu* ATGs and purple star are *A. tauschii* ATGs. The Green to red color represents the low to high bootstrap value. All identified ATGs were grouped into 19 clusters (subfamilies).

We identified 77, 51, 29, and 30 ATGs in wheat, wild emmer, *T. urartu,* and *A. tauschii*, respectively. These ATGs were grouped into 19 subfamilies according to the conserved motif and phylogenetic relations. ATG8, ATG13, ATG14, and ATG18 were the top four large subfamilies (Figure 1). On the whole, the number of *ATGs* across the species was consistent with the genome ploidy. The number of *ATGs* did not strictly follow the ploidy ratio (3:2:1) (Table S1: Additional file 1), possibly due to gene loss in hexaploidy wheat and intrachromosomal gene replication in diploid wheat. For example, the ATG8 subfamily had a significant expansion in tetraploid wheat species, in which it had seven members and was expanded to six chromosomes (1B, 2A, 2B, 5A, 5B, and 6A). However, ATG8 subfamily members in the 1B chromosome were lost in hexaploid wheat during the natural hybridization between wild emmer wheat and *A. tauschii.* The loss may be caused by an imbalance of gene number proportion in the A, B, and D subgenomes. In addition, the number of *ATGs* in plant species might correspond to the ploidy number of the genome (Table 1) [28,29,30,31]. At the same time, members of the ATG8 and ATG18 subfamilies were expanding dramatically in all detected plant species and not limited by the ploidy of the genome, indicating their durable and indispensable functions.

Physical positions of *ATGs* across the four species are presented in Figure S1 and the gene duplication events in Figure S2. A total of 72 gene duplication events were identified in wheat, out of which 25, 20 and 21 *TaATGs* appertained to the A, B, and D subgenomes, respectively. Most of the *TaATGs* had three homologous copies, except for the following five *TaATGs*: *Ta4A_ATG1a*, *Ta6A_ATG2a*, *Ta7A_ATG2c*, *Ta2A_ATG10a* and *Ta2A_ATG101a*. Members of the ATG8 subfamily tended to form intrachromosomal gene replication. For instance, duplicated genes *Ta2A_ATG8a* and *Ta2A_ATG8b* were found in chromosome 2A and *Ta2B_ATG8d* and *Ta2B_ATG8c* in chromosome 2B (Figure S2A). There were 14 gene duplication events in wild emmer. The gene duplication events between *Td1B_ATG8a* and *Td6B_ATG8g,* which were in different chromosomes, might be caused by chromosome segment exchange in the B genome (Figure S2B). Furthermore, two and three intrachromosomal gene replications were detected in *T. urartu* and *A. tauschii*, respectively.

Physical property, subcellular localization, gene structure, and conserved motif analyzes suggest significant conservation within the ATG subfamilies (Table S1: Additional file 2, Figure S3). For example, the characteristics of the ATG1, ATG3, and ATG11 subfamilies members were similar within the subfamily. However, gene structure and motif varied markedly within the subfamily, indicating the potential functional differentiation and variation. The ATG18 subfamily members could be divided into three distinct clades. The physical property, subcellular localization, gene structure and conserved motif of this subfamily were diverse among different chromosomes. In addition, the exon four of *Ta3D_ATG22d* was split into three smaller exons.

There are a total of 260 putative *cis*-elements in the 1.5-kb promoter region of *ATGs* from the four species. Of these, 59 were associated with plant growth and development, 37 with biotic and abiotic stress response, and 51 with plant hormone response (Table S2). The *cis*-elements related to water response (ACGTATERD1, MYCCONSENSUSAT and MYBCORE), copper-response (CURECORECR), plant hormone response (WRKY71OS, DPBFCOREDCDC3 and LTRECOREATCOR15) and plant growth and development (CAATBOX1, CACTFTPPCA1, DOFCOREZM, EBOXBNNAPA, RAV1AAT, GTGANTG10, SORLIP1AT and POLLEN1LELAT52) were abundant in the promoter region of *ATGs*.

### 2.2. Functional Annotation and Enrichment Analysis of Autophagy-Related Proteins

**Figure 2:**
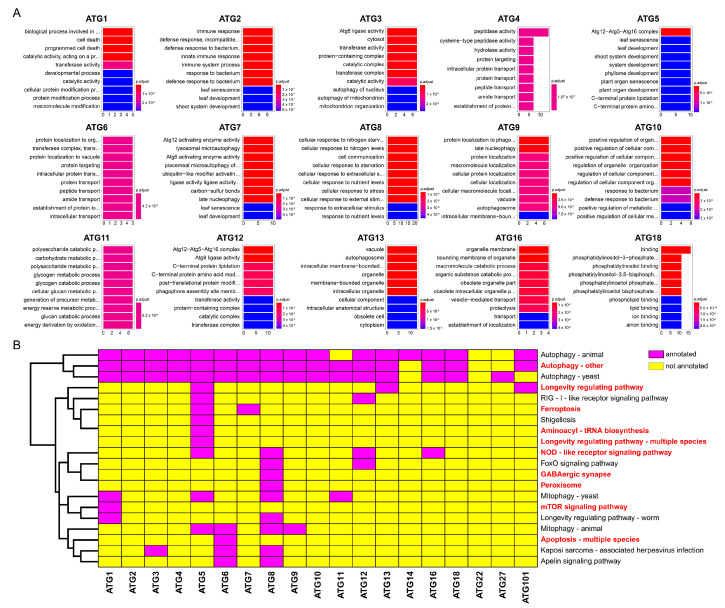
Annotation of identified ATGs. (**A**) GO enrichment of ATGs amongst different subfamilies. The top ten significantly enriched GO terms are displayed. The horizontal axis represents the number of ATGs which are annotated to the displayed GO terms; the vertical axis represents descriptive information of the enriched GO terms. Colors correspond to a value of *p*.adjust, which turns from blue to red as the *p*.adjust value changes from low to high. (**B**) KEGG enrichment of ATGs amongst different subfamilies. The horizontal axis represents autophagy subfamilies; the vertical axis represents descriptive information of annotated KEGG pathways. The fuchsia color denotes that the corresponding subfamily members were annotated in the KEGG pathway and the yellow color means that family members were not annotated. KEGG pathways highlighted by bold red font refer that these pathways can be found in plants.

The Gene Ontology (GO)enrichment results, performed according to annotation analysis of 187 ATGs, are shown in Figure 2A. As expected, different subfamilies hold diverse functions and play essential roles in various cellular life stages. The ATG2, ATG5, ATG6, ATG8, ATG10, and ATG11 subfamilies were mainly enriched in plant development, biotic and abiotic stress response, protein modification, transport and metabolic processes. Some autophagy-related subfamilies might work together on a particular biological function. For instance, the subfamilies ATG2, ATG5 and ATG7 were found to regulate leaf development and senescence and the ATG2 and ATG10 subfamilies might be responsible for plant resistance against bacteria. The functions of the ATG4 and ATG6 subfamilies were also similar to some extent.

Kyoto Encyclopedia of Genes and Genomes (KEGG) annotation implied that almost all autophagy-related subfamilies were annotated to the autophagy pathway except the ATG22 subfamily. No pathway was annotated in the ATG22 subfamily. Moreover, most of the ATG subfamilies participated in a specific cellular process. The ATG5 and ATG8 subfamilies held the most significant number of annotated pathways, indicating that these two subfamilies are crucial in the autophagy process. Our result implied that both the ATG5 and ATG7 subfamilies were annotated to ferroptosis, which is of potential interest for future studies on a simultaneous function of autophagy and ferroptosis (Figure 2B).

### 2.3. Protein-Protein Interaction Network of Autophagy-Related Proteins

**Figure 3:**
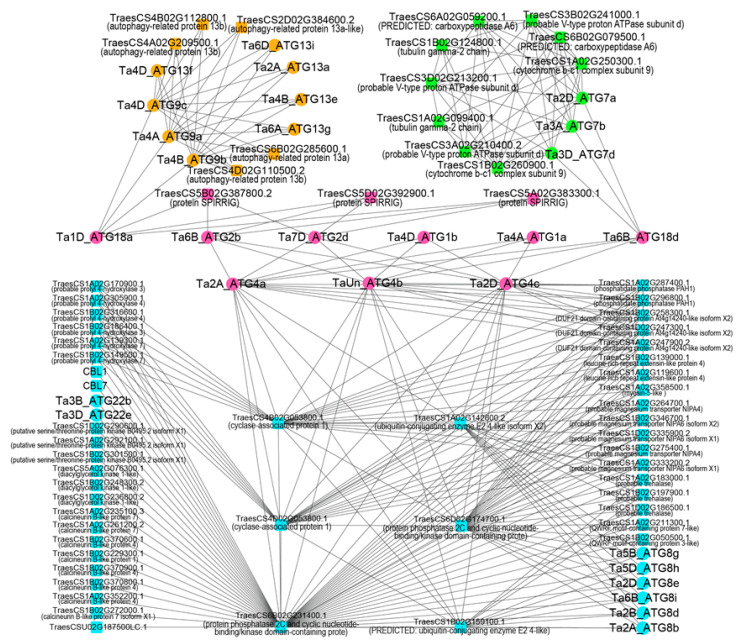
Protein-protein association networks of ATGs. The identified ATGs were submitted to the STRING database to construct the protein-protein interaction networks based on the *Triticum aestivum* dataset. The network could be clustered into four parts and represented in different colors. The circles are the ATGs identified in this study, and the squares represent the co-expressed proteins found in the database.

Protein-protein interaction relationships of ATGs in wheat can be divided into four clusters (Figure 3). In cluster I (cyan color), six ATG8 proteins and two ATG22 proteins interacted protein phosphatase 2C (PP2C) (*TraesCS6B02G231400.1*), a cyclic nucleotide-binding/kinase domain-containing protein. Three ATG4 proteins were at the node of the network. They not only interact with cyclase-associated protein 1, PP2C-associated genes and a ubiquitin-conjugating enzyme E2 4-like isoform for further indirect interaction with the ATG22 and ATG8 subfamily members, but also directly interacted with *Ta4A_ATG1a*, *Ta4D_ATG1b*, *Ta6B_ATG2b*, *Ta7D_ATG2d* and *Ta6B_ATG18d*. Our results implied that the protein SPIRRIG could interact with *Ta6B_ATG2b*, *Ta7D_ATG2d* and *Ta1D_ATG18a*, indicating that it may have participated in the autophagy process. In addition, both *Ta6B_ATG18d* and *Ta1D_ATG18a* served as bridges between ATG4 and ATG7 or ATG9 subfamily members, respectively; three ATG9 subfamily members were closely co-expressed with ATG13 related proteins.

### 2.4. Synteny Events and Selection Pressure Analysis of ATGs in Wheat and Its Diploid and Tetraploid Progenitors

**Figure 4:**
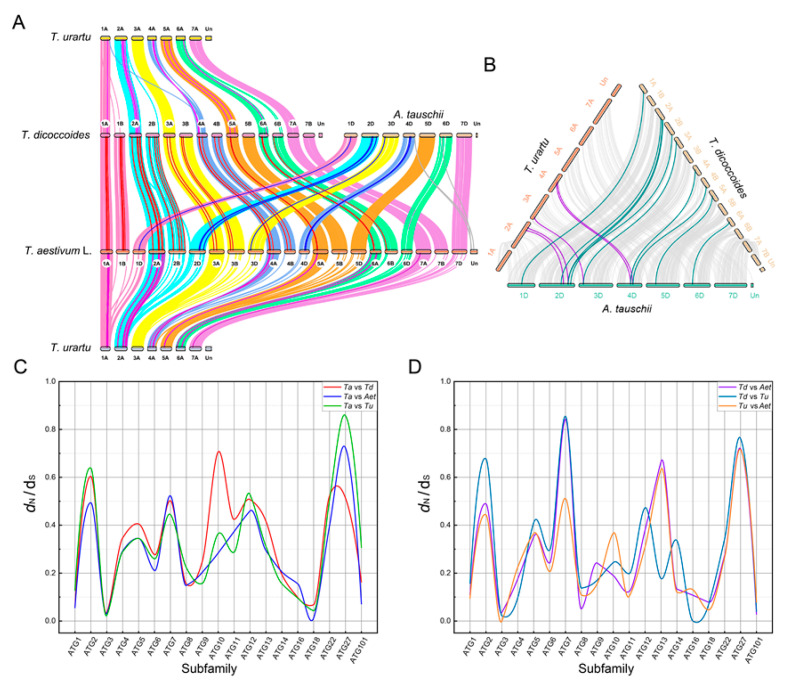
Collinearity relation of four Triticeae species and synonymous (*dS*) to nonsynonymous (*dN*) substitution ratio among ATG subfamilies. (**A**) Collinearity relations of *ATGs* between every two species are shown in different colors. (**B**) The collinearity relationship among *A. tauschii* to *T. urartu* and wild emmer. (**C**,**D**) The *dN/dS* ratio distribution of wheat and its three diploid and tetraploid progenitors among different subfamilies.

Synteny events were identified as markers for the relationship between ATG gene expansions and wheat polyploidization (Figure 4A,B). The number of syntenies between wheat and wild emmer is the largest (28) and the smallest between *A. tauschii* and *T. urartu* (5). Some syntenies among *ATGs* that belong to the same subfamily have shown a high homology during wheat evolution. For example, *Ta2A_ATG13a*, *Td2A_ATG13a*, *Tu2A_ATG13a*, and *Aet2D_ATG13a* were collinear, indicating that these genes are evolutionarily conserved and necessary. Furthermore, our results also suggest those synteny events in intrachromosomal repeat sequences. *Tu6A_ATG5a* and *Tu6A_ATG5b* were both collinear with *Ta6A_ATG5b*, and the reason for this collinearity may be that this gene duplication event occurred in *T. urartu,* but has been lost during the wheat hexaploidization process (Table S3).

Since a high *dS* value may imply a potential sequence saturation and misalignment [32,33], orthologous ATG gene pairs with *dS* > 0.3 were discarded and the remaining genes were further analyzed (Table S4). The highest average *dN/dS* value was recorded between wheat and wild emmer (0.35) and the lowest between wild emmer and *T. urartu* (0.26). The ATG subfamilies might be under various degrees of selection pressure during the evolution of wheat (Figure 4C,D). The ATG1, 3, 8, 18, and 101 subfamilies have experienced a stronger purifying selection pressure (*dN/dS* < 0.2) during the wheat evolution process. In addition, the selection pressure of the ATG10 subfamily between wheat and wild emmer was distinctly different, indicating that the nonsynonymous mutations of the ATG10 subfamily in wild emmer wheat tend to be retained in hexaploid wheat species.

An intense purifying selection was observed between wild emmer and *T. urartu* for the ATG16 subfamily. Fifteen *ATGs* were strongly negatively selected (*dN* = 0; *dS* ≠ 0) between wild emmer and wheat and eight *ATGs* were strongly negatively selected between *A. tauschii* and wheat. Moreover, only one strongly negatively selected *ATG* gene was detected in each of the following combinations: wheat vs. *T. urartu* (*Ta1A_ATG14a* vs. *Tu1A_ATG14a*), wild emmer vs. *A. tauschii* (*Td2B_ATG8c* vs. *Aet2D_ATG8a*) and wild emmer vs. *T. urartu* (*Td1A_ATG3a* vs. *Tu1A_ATG3a*).

### 2.5. The Single Nucleotide Polymorphisms (SNPs) Analysis of TaATGs

**Figure 5:**
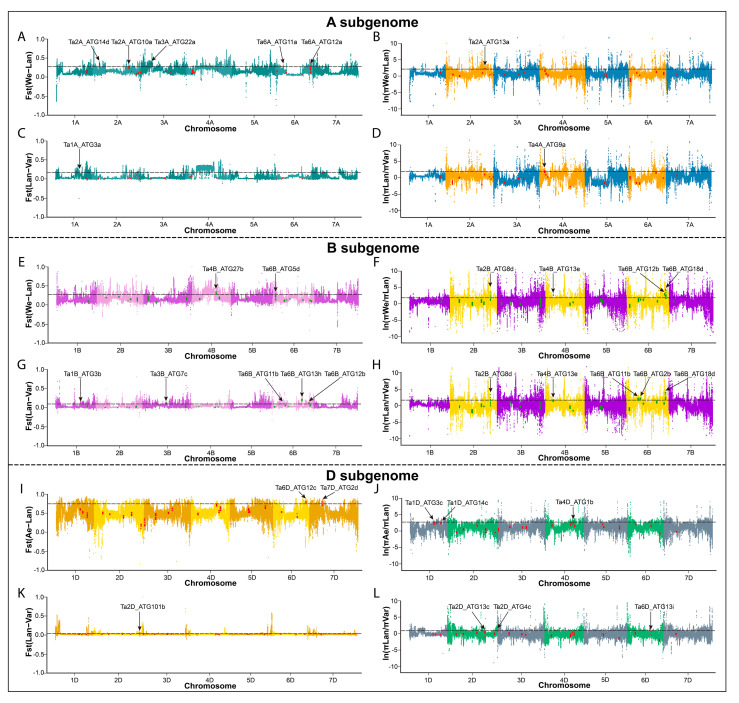
Distribution of *TaATGs* among genome-wide selective signals during domestication and breeding processes of wheat. (**A**,**B**,**E**,**F**,**I**,**J**) The *fst* and ln π ratios between wild emmer and a landrace were used to evaluate the domestication of wheat. (**C**,**D**,**G**,**H**,**K**,**L**) The *fst* and ln π ratio between a landrace and a variety were used to evaluate wheat’s improvement. Resequencing reads were mapped to the A,B and D subgenomes of wheat according to chromosome origin. All 77 *TaATGs* were highlighted against the position on each of the 21 chromosomes in different colors. The top 10% of the genome-wide value was taken as the threshold for selective sweeps and is shown as horizontal black dashed lines. The *TaATGs* above the threshold line are labeled.

The single nucleotide polymorphisms (SNPs) of *TaATGs* were analyzed across the 93 sequenced populations of wheat and its diploid and tetraploid progenitors (Table S5). No SNPs were detected in seven *TaATGs* localized in the promoter, exon, or intron regions, indicating indispensable roles of these *ATGs*. It is worth mentioning that SNP frequency within *TaATGs* varied amongst the A, B, and D subgenomes. *ATGs* located in the B subgenome held the most significant number of SNPs. Furthermore, π, *fst* and π ratio of all *TaATGs* were combined to analyze the evolution of *ATGs* during wheat’s domestication and improvement process (Figure 5, Figures S4–S6). In the A subgenome, six domestication-related and three improvement-related candidate *TaATGs* were found. For example, *Ta7A_ATG2c* has been identified as a candidate improvement-related gene and its π value for landrace was higher than that of other varieties, indicating that this gene might be selected during the improvement process of hexaploid wheat. There were six wheat domestication-related candidates and ten wheat improvement-related *ATGs* found in the B subgenome. *Ta2B_ATG8d*, *Ta4B_ATG13e*, *Ta6B_ATG12b*, and *Ta6B_ATG18d* were outstanding both in domestication and in improvement-related genes. In addition, five domestication-related and four improvement-related *TaATGs* were in the D subgenome. Nonsynonymous mutation of *ATGs* might represent essential selection effectors during wheat’s domestication and improvement process. As shown in Figures S7 and S8, nonsynonymous mutations were also found in *ATGs* located in A, B, and D subgenomes during the evolution of wheat.

### 2.6. Correlation Analysis of the Variations at the Ta2A_ATG8a Locus with the Seed Size of Tetraploid Wheat

**Figure 6:**
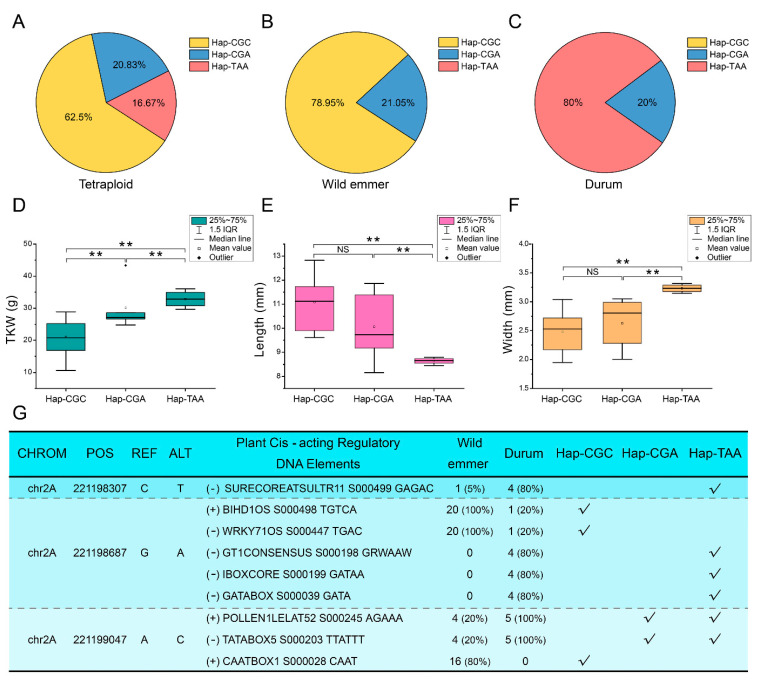
Haplotype-based phenotyping analysis. (**A**–**C**) Distribution of three haplotypes (Hap-CGC, Hap-CGA and Hap-TAA) in different tetraploid wheat populations. Three haplotypes located in the promoter region of *Ta2A_ATG8a* were found and their distribution in different tetraploid wheat populations was diverse. (**D**–**F**) Haplotype-based grain character analysis. The association between three haplotypes and grain characters was analyzed. Abnormal value of TKW, corresponding to HAP-CGA, was not considered in testing for significant difference test. ** *p* < 0.01. (**G**) Changes of *cis*-acting elements resulted from SPNs in the promoter region of *Ta2A_ATG8a* and their distribution in different populations.

DNA polymorphism assays found that tetraploid wheat accessions could be separated into three haplotypes (Hap-CGC, Hap-CGA, and Hap-TAA) based on three *cis*-acting elements changing SNPs located in the promoter region of *Ta2A_ATG8a* (Figure 6A–C). Four *cis*-acting elements, GAGAC, GRWAAW, GATAA, and GATA, were specific for the Hap-TAA accessions. Both AGAAA and TTATTT *cis*-acting elements were common in Hap-CGA and Hap-TAA accessions. Furthermore, three *cis*-acting elements, TGTCA, TGAC and CAAT, were specific to Hap-CGC accessions (Figure 6G). Haplotypes of wild emmer and durum were different: Hap-CGC and Hap-TAA were distinct and dominant for wild emmer and durum, respectively, while Hap-CGA was shared between them with similar proportions. The tetraploid wheat accessions with Hap-TAA held the highest thousand kernel weight (TKW) and grain width but shortest grain length. The TKW of Hap-CGA accessions was significantly higher than that of Hap-CGC. These results indicate that Hap-CGA represents a transitional stage during the tetraploid wheat domestication process. The Hap-CGA accessions with high TKW could have been preserved during the evolution from wild emmer to durum (Figure 6D–F). However, this association was not fully applicable to hexaploid wheat, indicating that the seed size of common wheat might be determined by the synergistic effect of multiple genes in the A, B, and D subgenomes. Moreover, the TKW was found to be significantly higher in hexaploid cultivar than in landrace (Figure S9), suggesting this parameter is a critical trait strongly selected for during the improvement process of hexaploid wheat.

### 2.7. Effects of Overexpression TaVAMP727 in Arabidopsis

**Figure 7:**
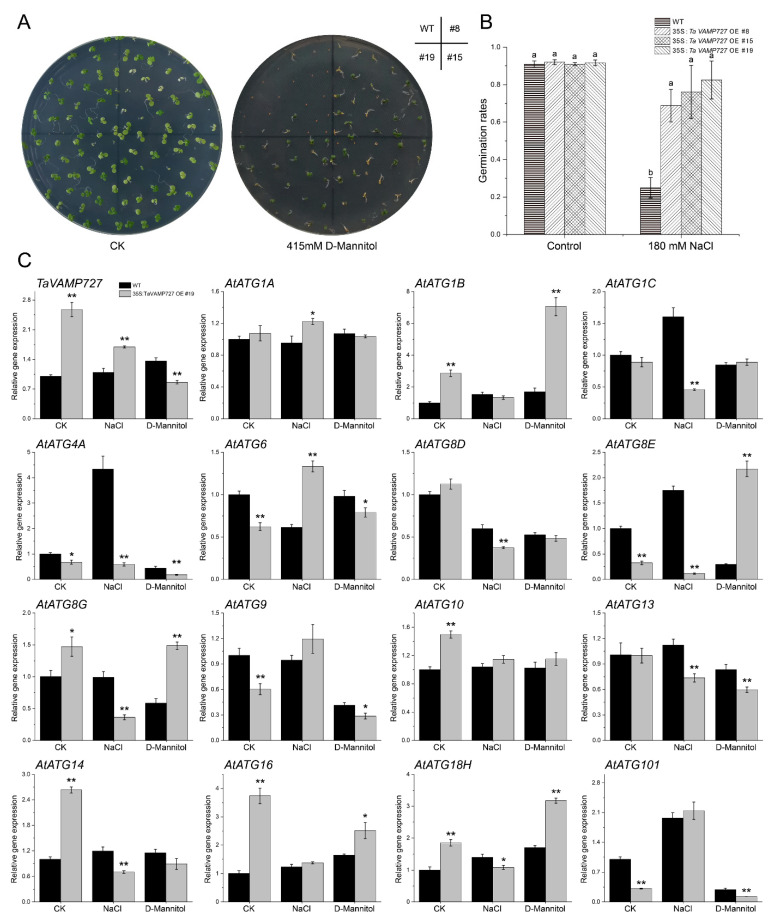
Influence of *TaVAMP727* on *ATGs* in *Arabidopsis*. (**A**,**B**) The germination rate of *TaVAMP727* overexpressed lines under either drought (415 mM D-mannitol) or salt (180 mM NaCl) stress. Error bars represent the SD. Columns bars followed by different letters are statistically different according to the analysis of variance followed by Duncan’s Multiple Range Test (control, *p* = 0.84; 180 mM NaCl, *p* = 0.0018; n = 3). (**C**) The expression levels of overexpressed *TaVAMP727* and 15 *Arabidopsis ATGs* were analyzed via reverse transcription-quantitative real-time PCR (RT-qPCR) in both wild-type and *TaVAMP727* transgenic line #19 under salt and drought stress. The ubiquitin 5 (*UBQ5*) (*At3G62250*) was used as housekeeping genes. Error bars represent the SD. * *p* < 0.05; ** *p* < 0.01; n = 3.

The VAMP727 is a seed plant-specific R-SNARE that may be crucial in autophagy [15,16,34]. *TaVAMP727* was cloned from wheat and then over-expressed in *Arabidopsis* to investigate its effects on autophagy and abiotic resistance. Seed germination showed that overexpressed *TaVAMP727* could significantly improve the germination rate of *Arabidopsis* under both drought and salt conditions (Figure 7A,B). The reverse transcription-quantitative real-time PCR (RT-qPCR) analysis revealed that *TaVAMP727* expression was significantly up-regulated under salt (Na^+^) stress but down-regulated under drought (D-mannitol) conditions in the transgenic line. As shown in Figure 7C, the expression profile of 11 *AtATGs* was significantly regulated by the overexpression of *TaVAMP727* under controlled conditions. *TaVAMP727* significantly up-regulated *AtATG1A and AtATG6* and down-regulated another nine *AtATGs* under salt stress. Additionally, *TaVAMP727* significantly up-regulated five *AtATGs* under drought stress and significantly down-regulated the other five *AtATGs*. These results indicated that the overexpression of *TaVAMP727* improved both salt and drought tolerance of *Arabidopsis* by regulating the expression levels of *ATGs*.

### 2.8. Effects of Overexpression TaVAMP727 in Wheat

**Figure 8:**
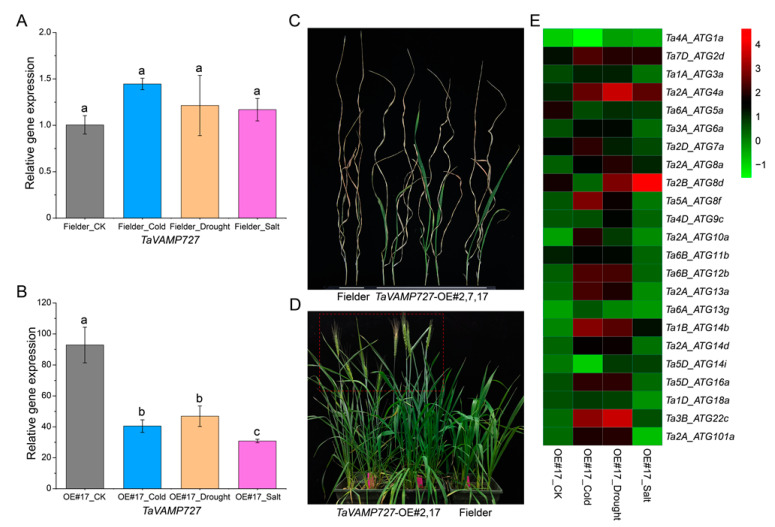
Overexpression of *TaVAMP727* in wheat. (**A**,**B**) The expression level of *TaVAMP727* in overexpressed line and Fielder (WT) under cold (4 °C) for 12 h, air-dry for 6 h and salt (150 mM NaCl solution) for 24 h. Error bars represent the SD. Columns bars followed by different letters are statistically different according to the analysis of variance followed by Duncan’s Multiple Range Test (A, *p* = 0.1947; B, *p* = 0.0005; n = 3). (**C**) Survival rate determination of trileaf stage Fielder and *TaVAMP727* transgenic wheat seedlings under drought stress. (**D**) The heating date comparison of Fielder and *TaVAMP727* transgenic lines. (**E**) The relative expression levels of 23 *TaATGs* in *TaVAMP727* overexpressed line under diverse abiotic stresses. The glyceraldehyde-3-phosphate dehydrogenase (GA3PD) was used as housekeeping genes.

The results gained from *Arabidopsis* might do not fully apply to wheat. Therefore, *TaVAMP727* was also overexpressed in wheat to ascertain whether *TaVAMP727* can regulate the expression levels of *ATGs* and further improve the abiotic stress resistance of wheat. RT-qPCR analysis showed that *TaVAMP727* was significantly overexpressed in transgenic lines under normal conditions. While under various abiotic stresses, including cold, drought and salt, it was significantly down-regulated, although it is still expressed at very high levels compared to Fielder at the same conditions (Figure 8B). In the non-transgenic wheat, the expression level of *TaVAMP727* was stable both under normal conditions and under abiotic stresses (Figure 8A). The transgenic wheat had a higher survival rate under drought stresses than that of Fielder; its heading date was at least six days earlier than that of Fielder (Figure 8C,D). The expression level of *TaATGs* was analyzed in transgenic wheat to explore the influence of *TaVAMP727* on autophagy. The results showed that the overexpression of *TaVAMP727* regulated the expression level of most *TaATGs* in response to the cold, drought and salt stresses. Most *TaATGs* in *TaVAMP727* transgenic wheat were up-regulated expressed under cold and drought stresses. The expression pattern of *TaATGs* under salt stress was different from that under cold and drought stresses (Figure 8E).

## 3. Discussion

An increasing number of studies have revealed that autophagy is crucial in plant development, stress response, senescence, and programmed cell death [5]. Polyploidy of a genome is accompanied by large chromosome segment loss, insertion, and rearrangement and these changes may lead to expression, silencing, or loss of genes [35]. Our study showed that two naturally interspecific hybridization events by three diploid donor species during the polyploidy process of wheat caused the unique landscape of the autophagy gene family among the wheat and its diploid and tetraploid progenitors (Table S1: Additional file 1). The number of *ATGs* was diverse among different subfamilies. The gene structures of *ATGs* were subfamily-specific, but within the subfamily, there were significant structural and functional differentiation (Figure S3). Selection pressures in wheat’s evolutionary process may have caused this within subfamily differentiations, since genes tend to evolve into diverse structures to fulfill multiple functions [36]. Prevalent structural divergences in duplicate genes can lead to the differentiation of functionally distinct paralogs [37]. Previous studies have shown that high wheat-specific inter- and intra-chromosomal gene duplications are potential sources of variability required for plant adaptation [38]. Gene rearrangement events, including both differential gene duplication and deletion within the A, B, and D regions, imply the rapid evolution of gene subfamilies after the separation of these three wheat subgenomes [39]. Our results support the view that gene duplication of *ATGs* came from homologous recombination, intrachromosomal replication and non-homologous chromosome exchanges during the hybridization process. This process can lead to environmental adaptation of wheat, although some homologous genes may be lost in the long evolutionary process.

Autophagy is a complex process that recruits a series of *ATGs* to perform different functions. In addition to its indispensable roles in the autophagy process, *ATGs* may also participate in other biological processes (Figure 2). Ferroptosis is a new form of cell death, first described in tumor cells and plant ferroptosis shares the main features of the process described in other systems [40,41]. Our results implied that both the ATG5 and ATG7 subfamilies were annotated to ferroptosis; thus, these two subfamilies may be related to wheat iron ion transport and are potential candidates for wheat quality breeding. In addition, ATG3, ATG5, ATG6, ATG7, and ATG8 subfamily members have been reported to affect plant immunity [42,43]. In this study, GO enrichment analysis showed that ATG2 and ATG10 subfamilies play essential roles in response to bacterial infection (Figure 2). Furthermore, we have identified that *ATGs* play critical functional roles in plant stress resistance and growth. ATG1, ATG2, ATG5, and ATG7 proteins were significantly annotated to the plant growth and development functions. The previous study has characterized the crucial role of *TdATG8* in drought and the osmotic stress response of wild emmer wheat [44]. Our results also revealed that ATG8 subfamily members of wheat and its Triticeae progenitors were indispensable to nitrogen starvation and abiotic stresses response. The interplay between phytohormones and multiple stresses is ubiquitous in plants [45]. Numerous *cis*-element of abscisic acid (ABA), auxin (IAA) and gibberellin (GA), as well as the *cis*-element of ethylene (ET), jasmonic acid (JA), and salicylic acid (SA) and cytokinin (CTK), were found concentrated in the promoter region of the identified *ATGs*.

Our results indicated that ATG4 could interact with ATG8 (Figure 3). The finding is consistent with previous studies in *Arabidopsis* [46]. In yeast, ATG4 proteolytic activity can be inhibited by ATG1 phosphorylation [47]. The interaction between *TaATG4* and *TaATG1* found in the present study suggest that this inhibition may also exist in plants. In *Arabidopsis*, protein SPIRRIG is essential for salt stress tolerance and endosomal transport routes [48,49]. Our interact network showed that SPIRRIG interacted with the ATG2-ATG18 complex further along with ATG9 to deliver lipids to the expanding phagophore, possibly improving the salt tolerance of wheat. In addition, close interactions between ATG9 and ATG13 were identified in our network. As a part of the ATG1/ATG13 kinase complex, dephosphorylated ATG13 interacted with ATG9 to stimulate lipids delivery [3]. PP2C has been reported as crucial in autophagy initiation and multiple abiotic stress responses [50,51]. Our results suggest PP2C may also be crucial in the crosstalk among *TaATG4*, *Ta6B_ATG18d*, *TaATG7*, and *TaATG22* (Figure 3).

Single nucleotide mutations play an important role in environmental adaptation [52]. In this study, we have identified nonsynonymous mutations of *ATGs* that may have been selected for the evolutionary process of wheat (Figures S7 and S8). In *Arabidopsis,* overexpression of ATG8 can increase nitrogen remobilization efficiency and improve grain filling significantly [53]. In our study, the *cis*-element variation resulting from DNA polymorphism in the promoter region of *Ta2A_ATG8a* is closely associated with TGW of tetraploid wheat (Figure 6). Wheat GAGAC *cis*-acting element has been reported to confer sulfur (S) deficiency response in *Arabidopsis* roots and S limiting can decrease wheat grain size [54,55]. The variation of the GAGAC *cis*-acting element in the promoter region of *Ta2A_ATG8a* may be responsible for the difference in grain size between wild emmer and durum wheat. In addition, the TTATTT *cis*-acting element has been reported to associate with the glutamine synthetase gene, which is crucial both for seed germination and for seed yield structure in *Arabidopsis* [56,57]. Thus, these two seed size-related *cis*-elements are prime candidates for selection in breeding tetraploid wheat.

Autophagy is an intracellular material circulation pathway that delivers intracellular material to the plant vacuoles. The final step in this process is the fusion of autophagosomes with vacuoles, which requires SNARE proteins [10]. Exploring the function of SNARE complexes in autophagy is central to understanding fusion processes and autophagy regulation. VAMP727 is a seed plant-specific R-SNARE that was vital in plant growth, development and defense [15,58,59]. Our research overexpressed the *TaVAMP727* in *Arabidopsis* and wheat to explore its roles in autophagy and plant growth (Figure 7 and Figure 8). Results showed that the overexpression of *TaVAMP727* can improve the abiotic stress resistance of plants by regulating the expression level of *ATGs* and promoting the heading of wheat. The overexpression of *TaVAMP727* may promote SNARE-mediated fusion of the autophagosomes with the tonoplast downstream of the autophagy process, which would accelerate the circulation of intracellular material and energy. Furthermore, autophagic homeostasis in cells might be disrupted by promoting autophagy in the later stage, and *ATGs* may change their expression level to maintain intracellular homeostasis, especially under stress conditions.

In summary, we have identified *ATGs* in wheat and its diploid and tetraploid progenitors systematically and characterized the landscape of the autophagy gene family in this study. We have also shown that overexpression of *TaVAMP727* improved the cold, drought and salt stress resistance of the transgenic wheat. Our findings demonstrate how autophagy genes regulate wheat plant development and its improved resistance to abiotic stresses, thus opening the route for transgenic wheat to potentially expand its range into colder, drier, and saltier areas.

## 4. Materials and Methods

### 4.1. Identification of ATGs in Wheat and its Diploid and Tetraploid Progenitors

Newly published protein sequences of wheat (*T. aestivum* L.) and wild emmer (*T. turgidum* ssp. *dicoccoides*) were downloaded from the Ensembl Plants database (http://ftp.ensemblgenomes.org/pub/plants/ (accessed on 1 January 2021)), *T. urartu* and *A. tauschii* genomes were downloaded from the NCBI (https://www.ncbi.nlm.nih.gov/genome/ (accessed on 1 January 2021)) and used to construct a local protein database. ATGs from different plant species were collected from NCBI (http://www.ncbi.nlm.nih.gov/ (accessed on 1 January 2021)) and earlier studies [16,28,29,30,31] and subfamilies of ATGs were then merged to construct an HMM profile using the ‘hmmbuild’ tool embedded in the HMMER3.0 web server (http://hmmer.org/download.html (accessed on 1 January 2021)). The ‘hmmsearch’ tool was further used to search for the ATGs with 1 × 10^−5^ as the threshold. In addition, all of the downloaded sequences were merged as a query to perform the local BLASTP search against genome sequences with e-value < 1 × 10^−5^ and identity > 60% as the threshold. The intersection proteins between HMM and the BLASTP search were considered as putative ATGs. Finally, redundant sequences were manually removed and one splice variant of putative ATGs was retained for further analysis. ATG domains were confirmed by PFAM (http://pfam.xfam.org/ (accessed on 6 January 2021)), NCBI Batch CD-search database (http://www.ncbi.nlm.nih.gov/Structure/bwrpsb/bwrpsb.cgi (accessed on 6 January 2021)) and InterProScan (http://www.ebi.ac.uk/interpro/ (accessed on 6 January 2021)) databases. For nomenclature, the prefix ‘*Ta*’, ‘*Td*’, ‘Tu’ and ‘*Aet*’ for wheat, wild emmer, *T. urartu* and *A. tauschii* were used separately and attached with chromosome information, followed by ‘ATG’. The serial number for each identified ATGs member was assigned according to their motif information. The online ProtParam tool (http://web.expasy.org/compute_pi/ (accessed on 6 January 2021)) was used to compute the grand average of hydropathicity (GRAVY), theoretical isoelectric point (pI) and molecular weight (Mw). The subcellular localization of each ATG protein was predicted using the online tool CELLO v.2.5 (http://cello.life.nctu.edu.tw/ (accessed on 6 January 2021)).

### 4.2. Bioinformatics Analysis

ATGs sequences were aligned to infer the unrooted phylogenetic tree using the IQ-TREE software with the bootstrap value estimation based on 1000 iterations and visualized by iTOL [60,61]. Chromosome locations of the identified *ATGs* visualized using Map Gene2Chromosome v2.0 (http://mg2c.iask.in/mg2c_v2.0/ (accessed on 10 January 2021)). The exon-intron structures were obtained from the annotation files and displayed using the Gene Structure Display Server website (http://gsds.cbi.pku.edu.cn/ (accessed on 10 January 2021)). Gene duplication events of *ATGs* were investigated and visualized by the Circos software [62,63]. The 1.5-kb genomic DNA sequences upstream of *ATGs* extracted from the genome data were then submitted to the online PLACE database (https://www.dna.affrc.go.jp/PLACE/?action=newplace (accessed on 10 January 2021)) to investigate putative *cis*-acting regulatory elements [64]. Conserved protein motifs were predicted by the MEME Suite (http://meme-suite.org/tools/meme (accessed on 15 January 2021)) using default parameters and visualized by the TBtools software [65]. The interaction network of identified ATGs from the four species was constructed using the STRING (v11) database (http://www.string-db.org (accessed on 20 January 2021)) based on the orthologous genes of wheat with the identity >50 as a threshold. All identified ATGs were annotated using the eggNOG database using the officially provided software ‘eggnog-Mapper’ with the DIAMOND algorithm [66]. The PAML_Yn00 tool was used to estimate the nonsynonymous (*dN*) and synonymous (*dS*) substitution rates of the ATG subfamily among four species [32]. The JCVI (v0.7.5) software was used to identify synteny genes based on the coding sequence (CDS) of four species [67].

### 4.3. The Single Nucleotide Polymorphisms (SPNs) Analysis of TaATGs Based on the Resequence Data

SNP information of *TaATGs* was obtained from 93 whole-genome resequencing data, including wheat and its diploid and tetraploid progenitors, adopted from Cheng et al. [25]. SNPs found in the promoter, mRNA, intron and exon regions of *TaATGs* were extracted according to the location data using the ‘bcftools’ software from the Samtools package [68]. Additionally, π and *fst* values were also calculated using the ‘vcftools’ software [69]. Seeds of the sequenced taxa were from our lab collection. Seed grain characteristics were measured using a Wanshen SC-G seed test instrument (Wanshen Testing Technology Co., Ltd., Hangzhou, China).

### 4.4. Genetic Transformation of Arabidopsis and Wheat

The coding region of *TaVAMP727* (*TraesCS7A02G279100.1*) was PCR-amplified using the primers F: 5′-CGCGGATCCATGAACGGTGGTAGCAAGC-3′ and R: 5′-GGGGTACCCTAGCACTTGAAGCCCCTG-3′ from the first-strand wheat cDNA and subsequently verified by Sanger sequencing. It was digested by BamHI and KpnI and ligated between the ED35S promoter and NOS-Ter (nopaline synthase terminator) in the binary vector pWR306 (transformed from pCAMBIA1303) [70]. This construct was introduced into *Agrobacterium tumefaciens* GV3101 and then transformed into *Arabidopsis thaliana* (Columbia) using the floral dip method [71]. Seeds of homozygous T3 lines and wild type (WT) were surface-sterilized and germinated on Murashige and Skoog medium (MS medium), MS medium supplemented with 400 mM D-mannitol and 180 mM NaCl for stress resistance analysis. Seeds of WT and *TaVAMP727* transgenic lines were then placed on MS medium, MS medium enriched with 120 mM NaCl or 200 mM D-mannitol for RT-qPCR analysis. All Petri dishes were put into a growth chamber under 16-h-light/8-h-dark cycles and 22 °C/19 °C day and night temperature, respectively, and grown for three weeks. Seedlings from each treatment were collected, immediately frozen in liquid nitrogen and stored at −80 °C for further use.

Total RNA was isolated using Plant RNA Kit (Omega Biotek, Norcross, GA, USA) and the integrity was checked on 1% agarose gels by staining with ethidium bromide. Furthermore, RNA amount and purity were quantified using a Nano Drop ND-1000 instrument (Nano Drop, Wilmington, DE, USA). The first-strand cDNAs were synthesized using Evo M-MLV Mix Kit with gDNA Clean for qPCR (AG11728, Accurate Biotechnology (Hunan) Co., Ltd., Hunan, China) with random primers. The RT-qPCR analysis was conducted using a StepOnePlus™ Real-Time PCR System (ABI, Carlsbad, CA, USA) with SYBR^®^ Green Premix Pro Taq HS qPCR Kit (Rox Plus) (AG11718, Accurate Biotechnology (Hunan) Co., Ltd., Hunan, China). Three replicates and three technical repetitions were used for each RT-qPCR experiment and the expression levels of 15 *Arabidopsis ATGs* were analyzed. The ubiquitin 5 (*UBQ5*) (*At3G62250*) were used as housekeeping genes [72]. Primers used in this analysis are presented in Table S6: Additional file 1. The expression level was calculated according to the 2^−ΔΔCT^ method [73].

The sequencing verified coding region of *TaVAMP727* digested by HindIII and EcoRI and ligated to plant binary expression vector pCAMBIA3301. This construct was transformed into Fielder using the *Agrobacterium tumefaciens*-mediated transformation method [74]. The specific detection primer pairs (F: 5′-TCGATGCTCACCCTGTTGTTTG-3′, R: 5′-TGTATAATTGCGGGACTCTAATC-3′) were used to validate the presence of *TaVAMP727* in the transgenic wheat. Seeds of T3 transgenic lines and Fielder were soaked in water, germinated at 25 °C for two days, and then transferred to a half-strength Hoagland’s liquid medium. Seedlings grown in the liquid medium were placed in a growth chamber under controlled conditions with 25 ± 1 °C, 16-h-light/8-h-dark cycles. Trileaf stage *TaVAMP727* transgenic seedlings and Fielder seedlings were treated with cold (4 °C) for 12 h, air-dry for 6 h, salt (150 mM NaCl solution) for 24 h and normal conditions. Seedlings of Fielder under normal conditions were used as a control to detect the response of *TaVAMP727* to cold, drought and salt in non-transgenic wheat. In addition, Fielder seedlings under normal, cold, drought and salt stresses were considered as a control for transgenic seedlings in corresponding conditions. All seedlings were collected at the stated time points and were immediately frozen in liquid nitrogen and stored at −80 °C for further use. RT-qPCR analysis was used to detect the influence of *TaVAMP727* on 23 *TaATGs* and glyceraldehyde-3-phosphate dehydrogenase (*GA3PD*) was used as housekeeping genes [75]. Methods of total RNA extraction, first-strand cDNAs synthesis, RT-qPCR and relative expression level calculation were the same as the described above. The primers used here are listed in Table S6: Additional file 2. In addition, Fielder and *TaVAMP727* transgenic wheat seeds germinated in the soil were grown in normal conditions. When seedling reached the trileaf stage, watering was stopped until wilting and then rehydrated to detect drought resistance.

### 4.5. Statistical Analysis

Statistical analysis was conducted by SAS (version 9.2), using an analysis of variance (ANOVA). The ANOVA means was tested by Duncan’s multiple range test at the 0.05 and 0.01 level.

## Figures and Tables

**Table 1:** The subfamily distribution of *ATGs* among different species.

Subfamily	*T. aestivum* L.	*T. dicoccoides*	*T. urartu*	*A. tauschii*	*O. sativa* L.	*S. italica* L.	*N. tabacum* L.	*M. acuminata*
ATG1	2	1	1	1	3	1	3	1
ATG2	4	3	2	2	0	1	1	1
ATG3	3	2	1	1	2	2	1	3
ATG4	3	2	1	1	2	1	1	3
ATG5	5	2	2	1	1	1	1	1
ATG6	2	1	1	1	3	2	1	1
ATG7	4	3	1	2	1	2	0	1
ATG8	8	7	3	3	7	4	5	10
ATG9	3	2	1	1	2	2	1	2
ATG10	2	2	1	1	2	1	1	0
ATG11	3	2	1	1	0	1	0	0
ATG12	4	3	2	3	1	1	0	1
ATG13	9	6	2	3	2	3	3	2
ATG14	9	3	3	1	0	0	0	0
ATG16	1	1	1	1	1	1	0	1
ATG18	4	5	3	4	6	7	6	5
ATG19	5	2	1	1	0	0	0	0
ATG20	0	0	0	0	0	0	1	0
ATG27	3	2	1	1	0	0	0	0
ATG101	2	2	1	1	0	0	0	0
all	76	51	29	30	33	30	25	32

## Data Availability

Not applicable.

## Supplementary Materials

The following are available online at https://www.mdpi.com/article/10.3390/ijms23020891/s1.
