# Supplementary material for: The Landscape of Autophagy-Related (ATG) Genes and Functional Characterization of TaVAMP727 to Autophagy in Wheat
Source: Int J Mol Sci. 2022 Jan 14;23(2):891. doi: 10.3390/ijms23020891 (PMC8776105; doi:10.3390/ijms23020891)
Supplement: Supplementary file 1 [file ijms-23-00891-s001.zip › Supplementary_Figures.pdf]

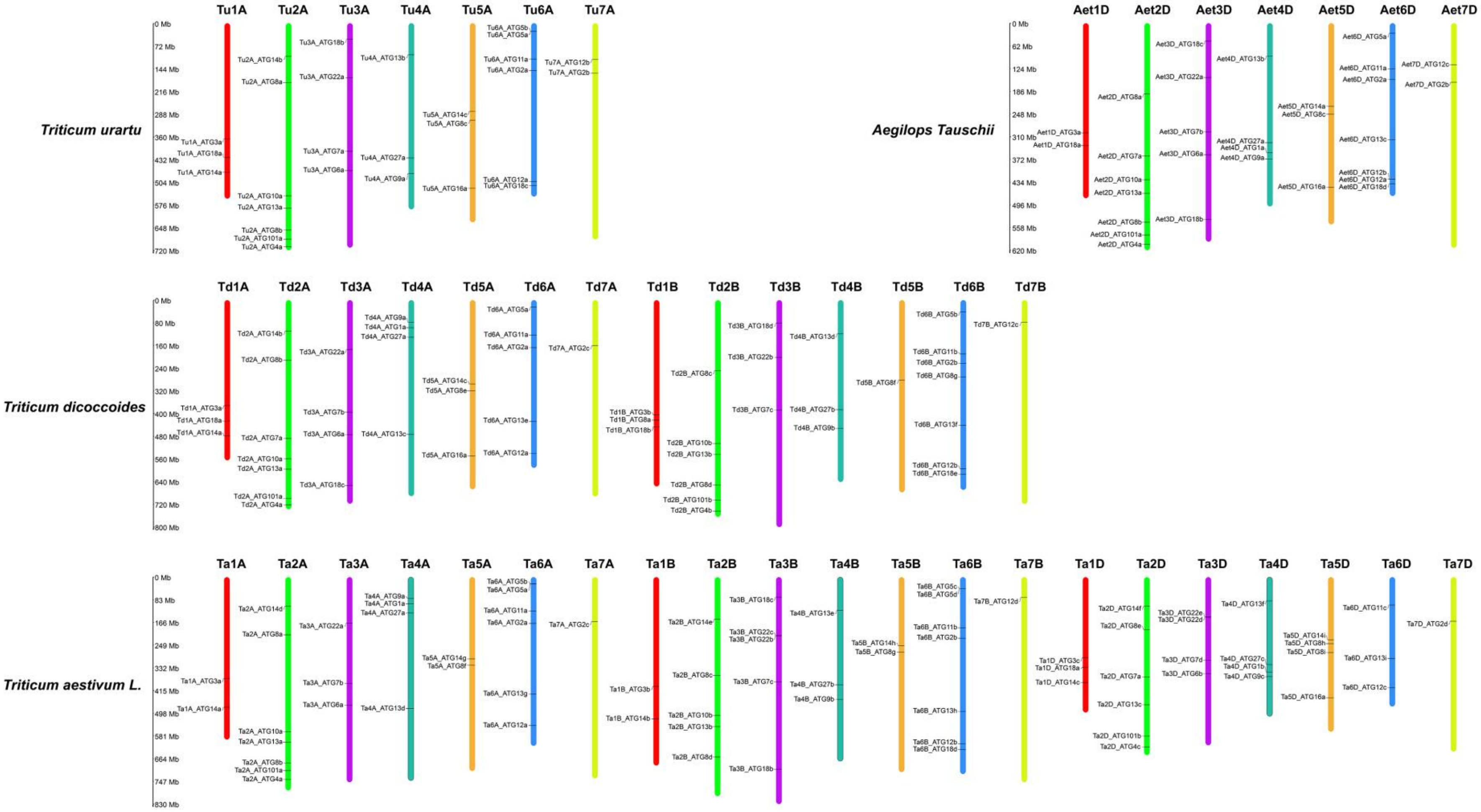

Figure S1. Distribution of *ATGs* along with the *Triticum aestivum* L., *Triticum dicoccoides*, *Triticum urartu* and *Aegilops tauschii* chromosomes. The chromosomal distances are given in Mb.

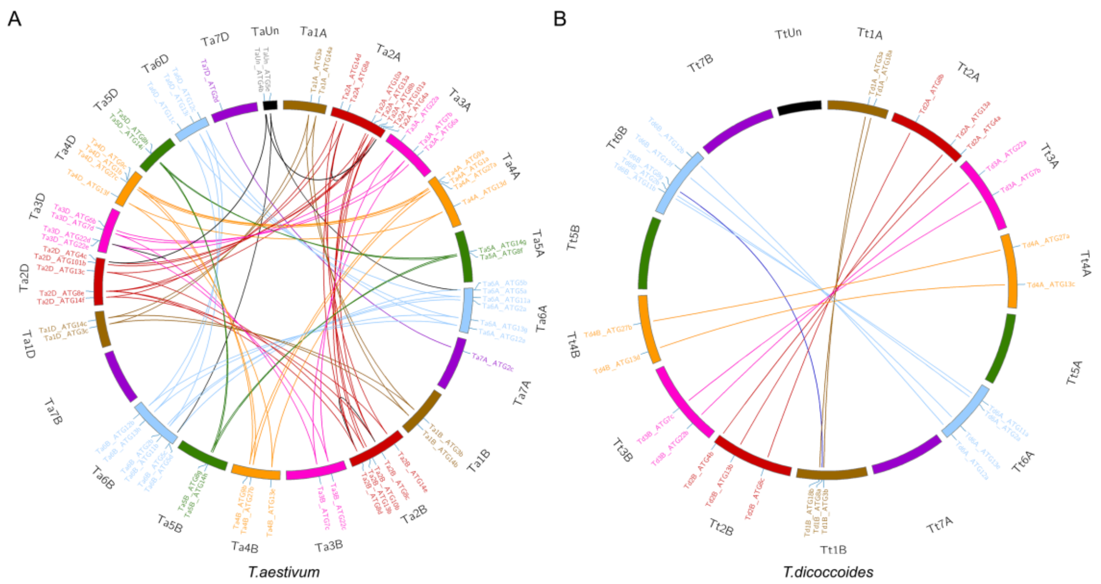

Figure S2. Gene duplication events of *ATGs* in wheat and wild emmer subgenomes. (A) Gene duplication events in wheat. (B) Gene duplication events in wild emmer. Sven homologous groups of wheat and wild emmer chromosomes are displayed in different colors. Duplicated genes of each homo-group are displayed and linked with the corresponding color. Furthermore, there were no gene duplication events found in *Triticum urartu* and *Aegilops tauschii*.

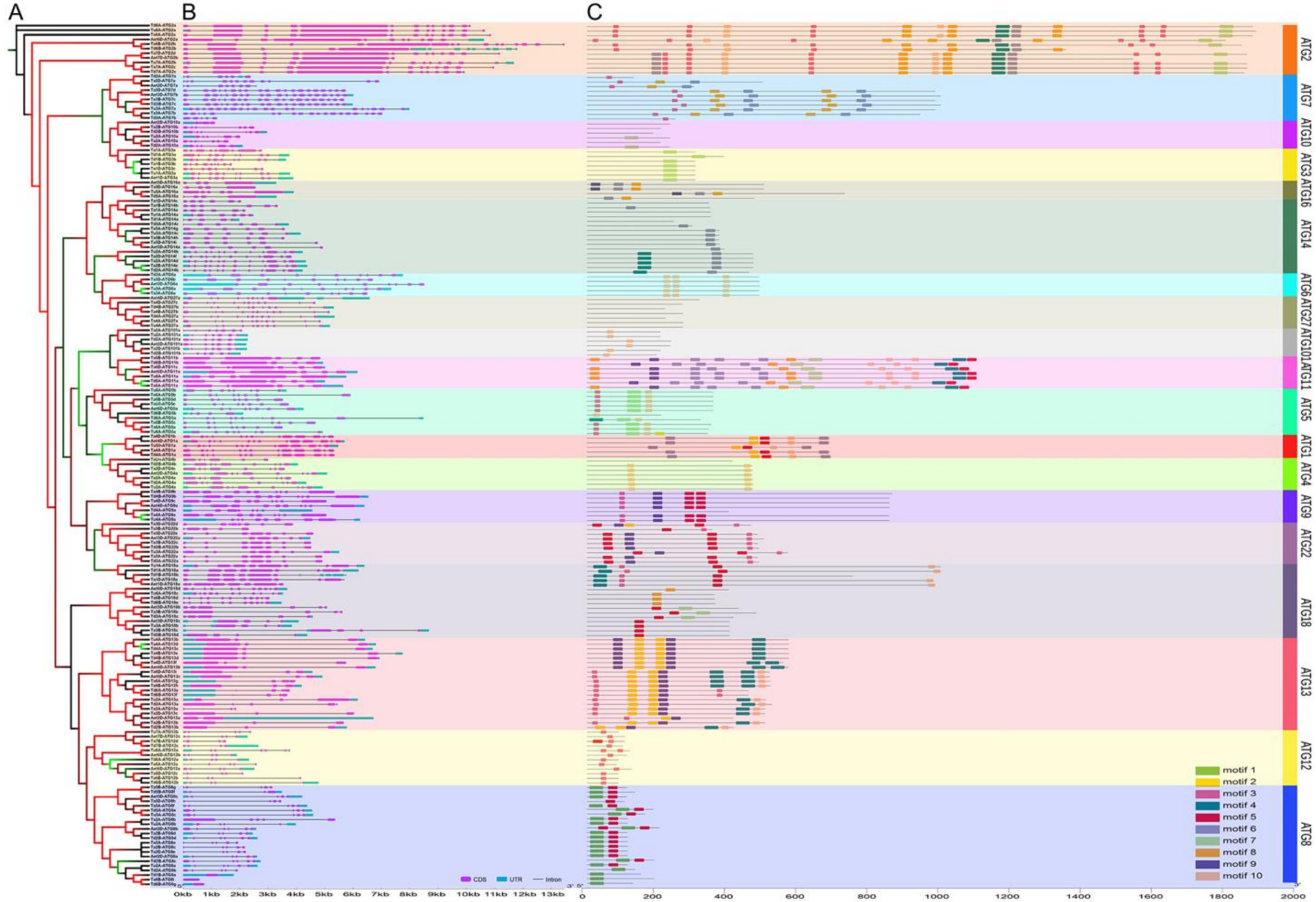

Figure S3. Phylogenetic relationships, gene structures and conserved motifs of all 187 *ATGs* in wheat and its diploid and tetraploid progenitors. (A) The phylogenetic relationships of all 187 *ATGs*. (B) The exon-intron structures of identified *ATGs* and displayed using the Gene Structure Display Server. (C) Conserved protein motifs of all *ATGs*.

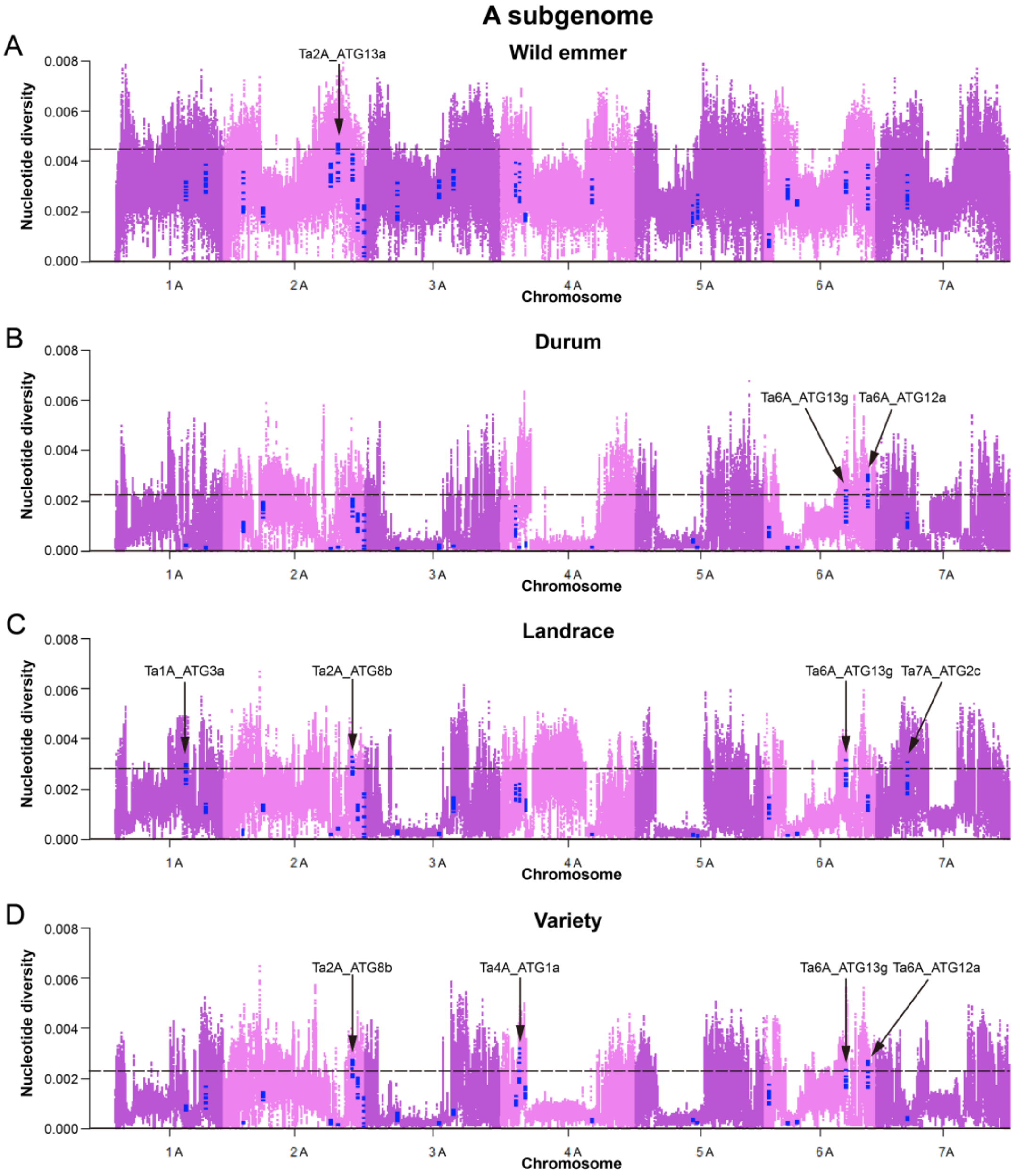

Figure S4. Nucleotide diversity (π) of the *TaATGs* in the A subgenome. (A) Wild emmer. (B) Durum. (C) Landraces. (D) Varieties. The π value was calculated in a non-overlapping 100 kb window. All *TaATGs* on A subgenome were highlighted against the position on each of the seven chromosomes in blue colors. The top 10% of the genome-wide value was taken as the threshold for selective sweeps and showed with horizontal black dashed lines and the *TaATGs* above the threshold line were labeled.

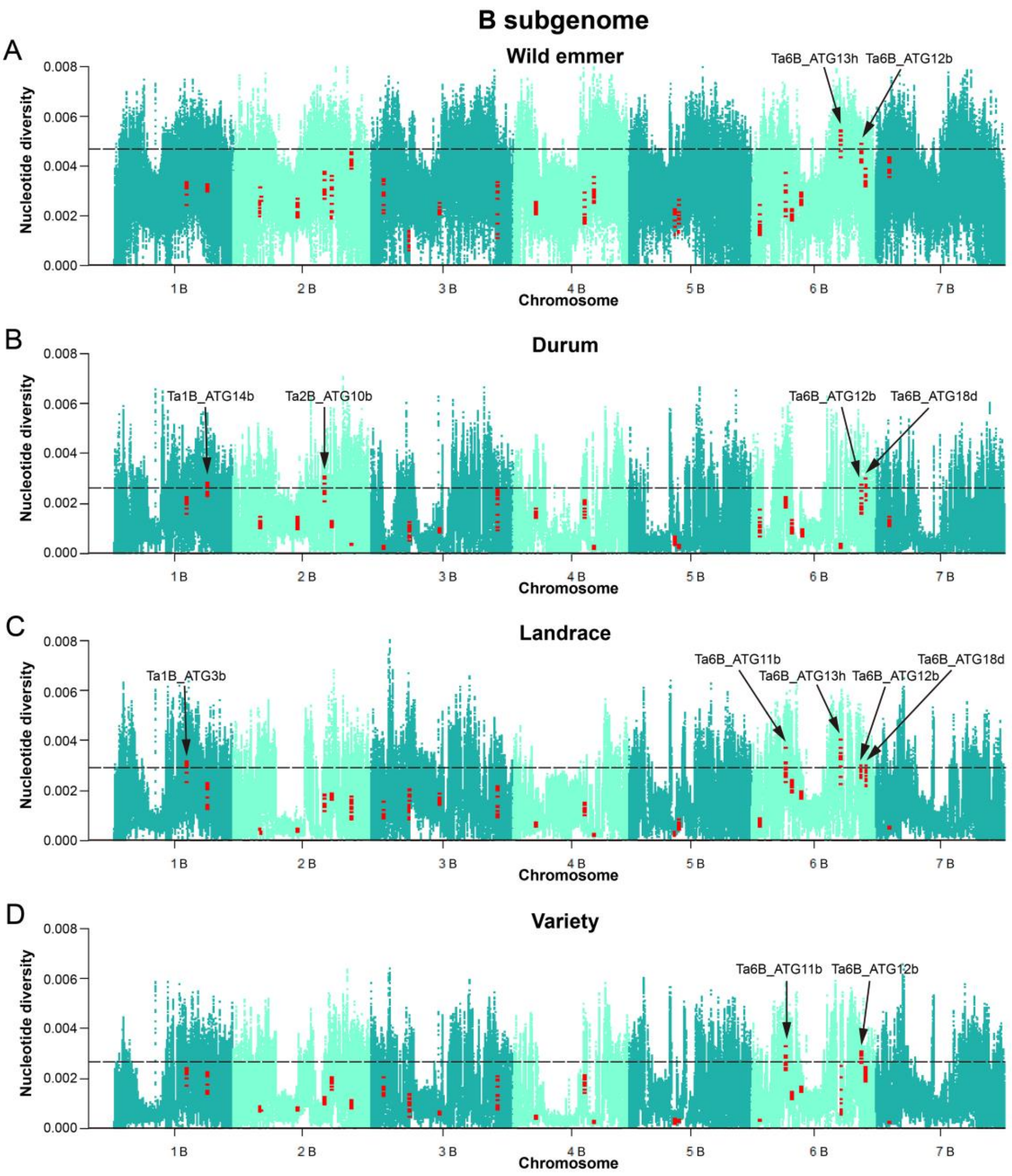

Figure S5. Nucleotide diversity (π) of the *TaATGs* in the B subgenome. (A) Wild emmer. (B) Durum. (C) Landraces. (D) Varieties. The π value was calculated in a non-overlapping 100 kb window. All *TaATGs* on the B subgenome were highlighted against the position on each of the seven chromosomes in red colors. The top 10% of the genome-wide value was taken as the threshold for selective sweeps and showed with horizontal black dashed lines and the *TaATGs* above the threshold line were labeled.

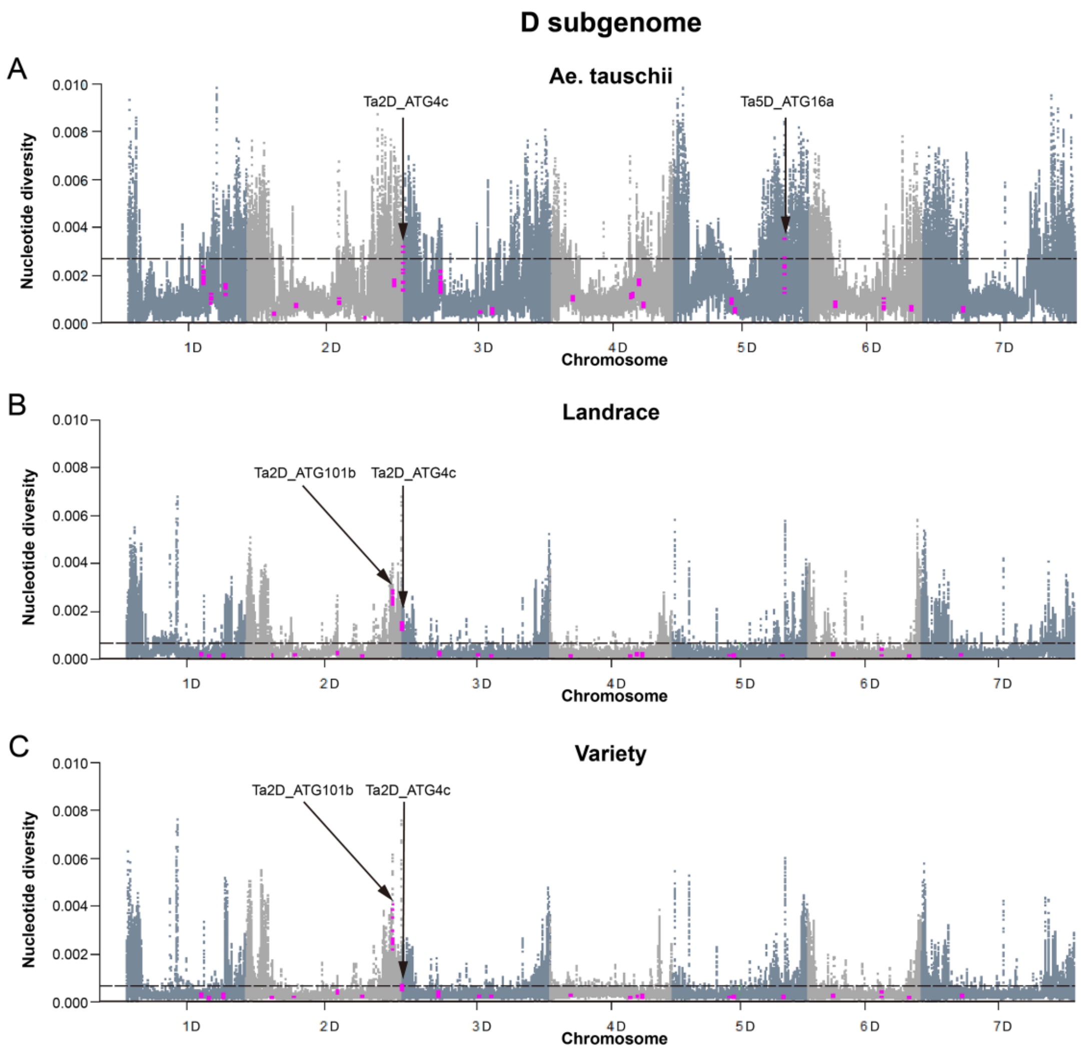

Figure S6. Nucleotide diversity (π) of the *TaATGs* in the D subgenome. (A) *Aegilops tauschii*. (B) Landraces. (C) Varieties. The π value was calculated in a non-overlapping 100 kb window. All *TaATGs* on the D subgenome were highlighted against the position on each of the seven chromosomes in purple colors. The top 10% of the genome-wide value was taken as the threshold for selective sweeps and showed with horizontal black dashed lines and the *TaATGs* above the threshold line were labeled.

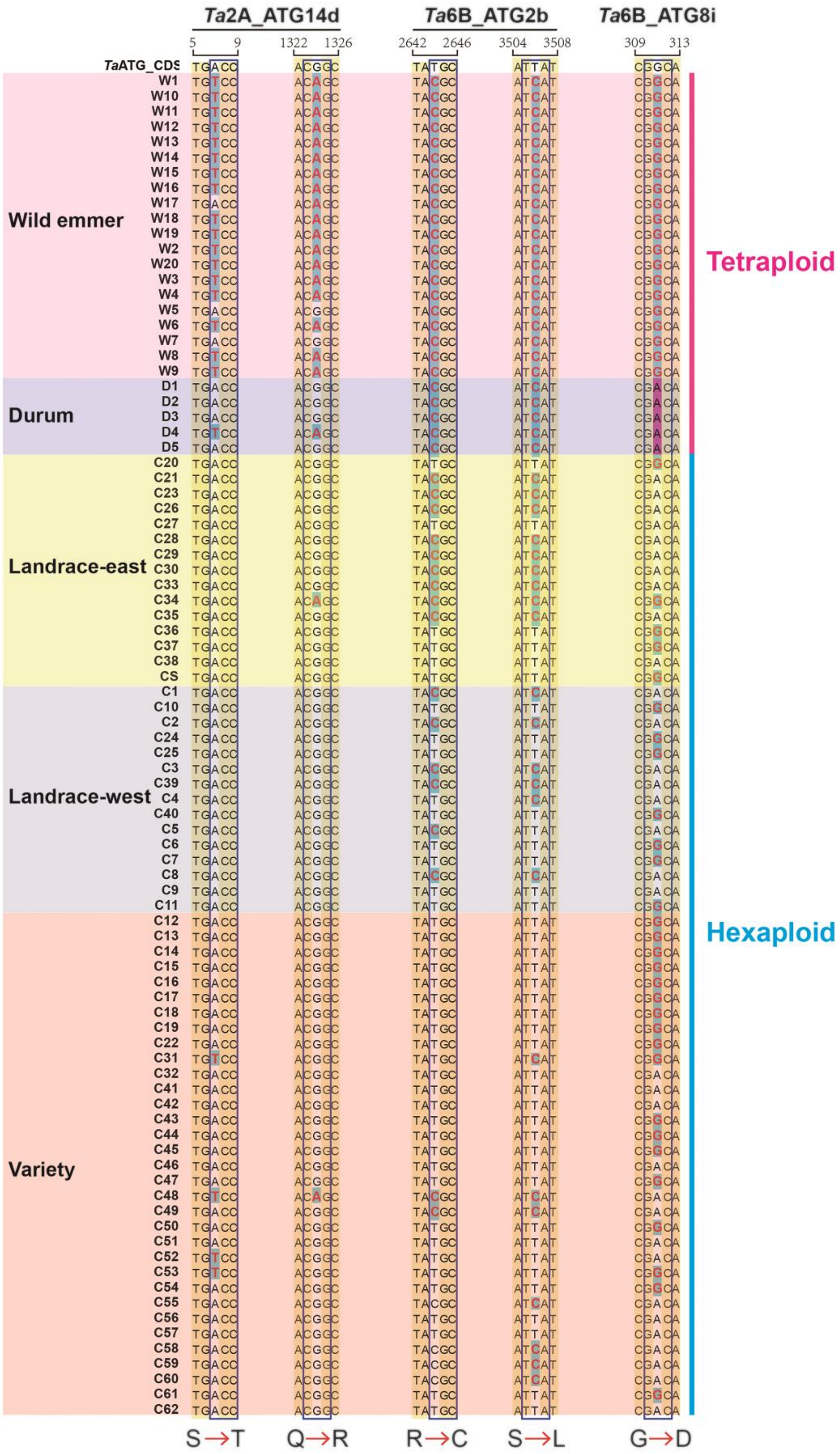

Figure S7. Selection of nonsynonymous mutations in *TaATGs* amongst various subpopulations in the A and B subgenomes.

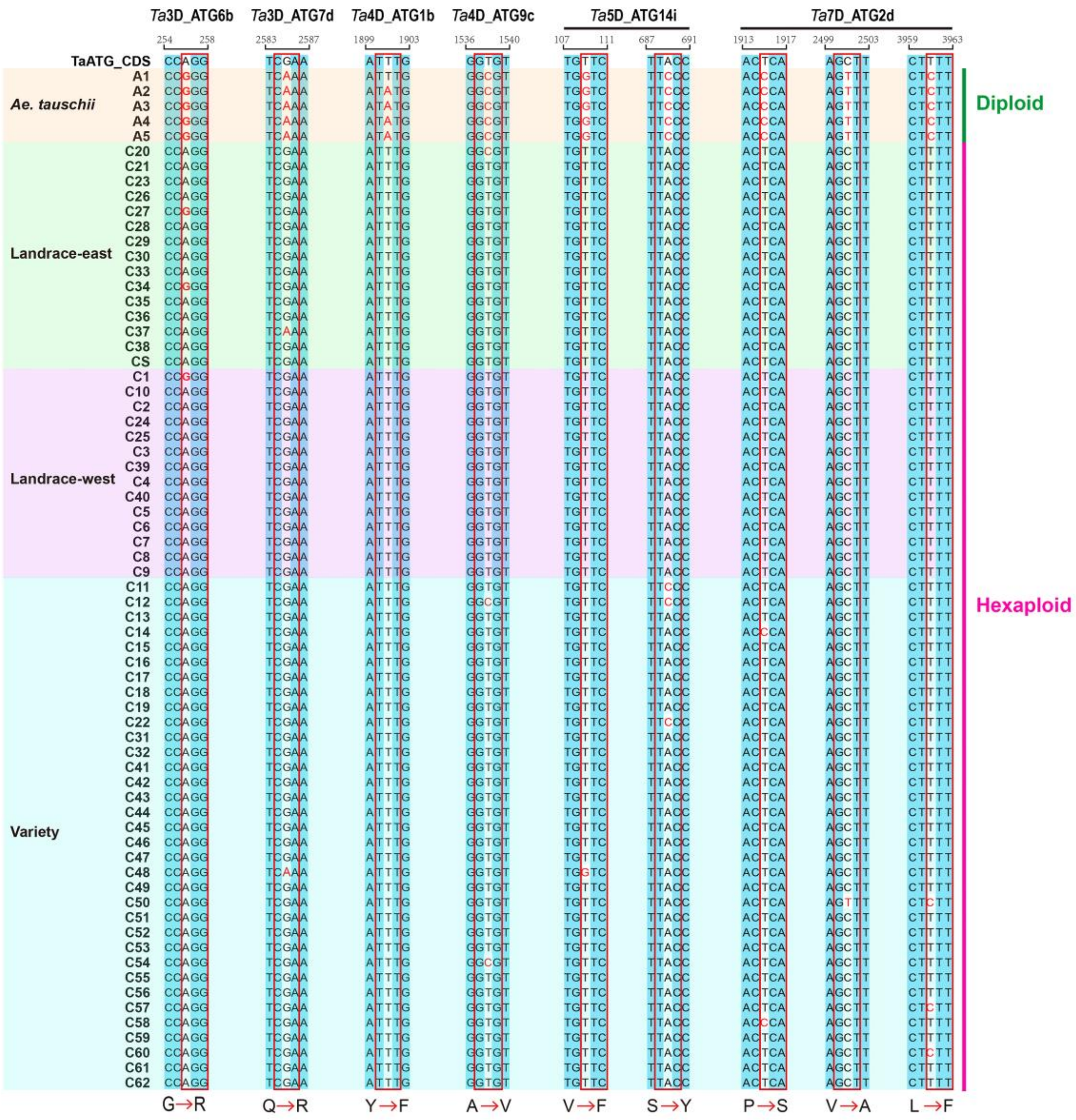

Figure S8. Selection of nonsynonymous mutations in *TaATGs* amongst various subpopulations in the D subgenomes.

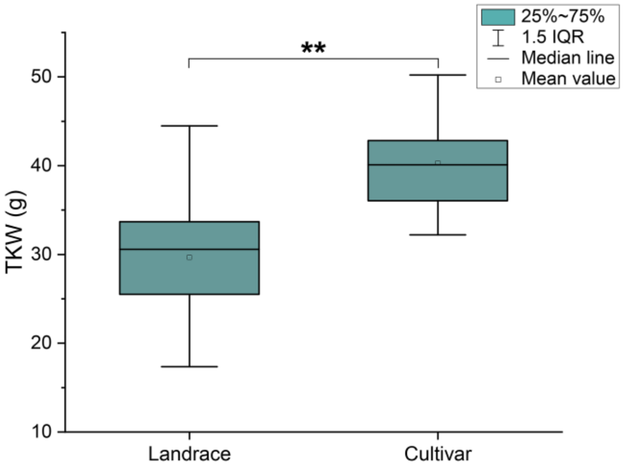

Figure S9. Thousand kernel weight (TKW) of hexaploid landrace and cultivar. **$P < 0.01$.
